# Mr. Ring, on Vaccination

**Published:** 1805-05-01

**Authors:** John Ring

**Affiliations:** New Street, Hanover Square


### *To the Editors of the Medical and Physical Journal.*

GENTLEMEN,

A Writer in the last number of the Medical and Chirurgical Review, says, he is glad Mr. Goldson has not been deterred from prosecuting his inquiries. Yet he acknowledges, that the new evidence offered by that gentleman is neither very extensive nor important. We may therefore conclude, that the Reviewer is pleased at seeing another impotent attempt to depreciate vaccination, and augurs a new triumph to the practice.

Mr. Goldson tells us in his preface, that his *veracity* has been called in question. It was natural, therefore, to suppose, that he would have vindicated his veracity; but after a careful perusal of his work, I have not been able to discover any such vindication.

The Reviewer notices Mr. Goldson's allusion to the case in Wilderness Lane,* which, he says, I deem a spurious cow-pock. This is what I have no where asserted. I gave my opinion, that the attempt to vaccinate proved abortive; and coincided in opinion with the officers of the Vaccine Pock Institution, that the child did not go regularly thro' the cow-pock.

It is commonly allowed, that a genuine cow-pock may disappear before the proper time; and, consequently, without affecting the habit. Instances of that sort were published in this Journal by Dr. Harrison; and are analogous to cases which have occurred, of local small-pox after inoculation. This idea did not suit Mr. Goldson's purpose. He has, therefore, been guilty of misrepresentation; as in the case of Mr. Grant's child, which I stated in my answer to his first pamphlet.

Had it been a spurious pustule, he thinks I might have been able to detect it, from my intimacy with Dr. Jenner. He is fond of writing on subjects which he does not understand; it is therefore necessary to inform him, that when this case occurred, I had never received much instruction from Dr. Jenner; who had then resided but little in London, since the promulgation of his discovery.

Mr. Goldson thinks, I must have been very soon admitted to a perfect knowledge of vaccination. I do not wonder

* See Medical and Physical Journal for February last, p. 175.

der at his deeming it an easy task to learn the practice, when he thinks, that one who knows so little of it as himself, is capable of writing on the subject.

He adverts to my offer of submitting my vaccine patients to the test; and pretends it was indiscriminate. The offer however was, as will be seen by any one who reads it, that "*if any gentleman, who has infected two, ten, or twenty of his own vaccine patients with variolous matter, will take the trouble of inoculating mine with the same, I will furnish him with at least a thousand, on whom he may exercise his skill.*" This is but a trifle, in a gentleman who has a talent for misrepresentation, like Mr. Goldson.

Another article in the Review, is a critique on Dr. Moseley's publication. The Reviewer has not noticed his want of candour, in confounding the cow-pock with the murrain; but he has noticed his reflections on those who sell cow-pock matter; whom he happily describes as *milking, selling the cream,* and converting the *cow* into *a golden calf.*

In a subsequent part of the Review is, what is called a Critical Examination of Mr. Goldson's Second Pamphlet. This is ascribed to Dr. Pearson. If, however, we may judge from intrinsic evidence, it cannot be his production; but, like the publication of the Bramin in Bengal, mentioned by Mr. Shoolbred, a base forgery.

It contains, to use the elegant language of that Review, a "*senseless outcry*" against the opponents of Mr. Goldson; between whom and the author of the critique, there seems to be a considerable degree of sympathy. In short, they appear, on this occasion, to shake hands most cordially; to pay each other a vast number of compliments, and to make one common cause.

When Mr. Goldson's first pamphlet appeared, we were told in the Review, that it was not to be answered by *arguments,* but by *facts.* No argument in favour of vaccination can be drawn but from facts. Yet this "*senseless outcry*" has been resounded from the Tweed to the Delaware; Editors of Journals and Reviews have admitted the fallacy at the suggestion of its author; and been the easy dupes of that imposition.

Much paper is wasted in proving, what I long ago proved, in my Answer to Mr. Goldson, that a local variolous pustule, in those who are put to the test after vaccination, is no proof of the inefficacy of the cow-pock. It is now also shrewdly discovered, that arguments may be adduced from Mr. Goldson's own evidence against his conclusions, clusions, without having recourse to hazardous experiments for that purpose.

We were told, indeed, in one publication, that certain gentlemen knew such experiments were perfectly safe; yet a proof of the contrary appeared in the next page. This is a very bad specimen of that "*medical logic*," for which the said curious production is so much puffed off in the Review.

By the Edinburgh Medical and Surgical Journal it appears, that Dr. Pearson is now convinced of the propriety of putting those, whose security against the small-pox is doubtful, to the test of vaccine rather than variolous inoculation; which, as the learned editors justly observe, is on so many accounts objectionable. It must give great pleasure to every friend of humanity to hear, that those formidable enemies of children, armed with their envenomed weapons, at length sound a retreat;

> And he, who erst had made a din most,
> Now cries, the devil take the hindmost.

In a subsequent part of the Critical Examination of Mr. Goldson's Pamphlet, we are told it is important to those who have answered that gentleman, to provoke the credit of a controversy. We are not told, however, what credit can accrue from a controversy with such an author, who, even according to this very Review, taxes his reader with three shillings, and the trouble of toiling through a dull and voluminous pamphlet for the sake of information, which is "neither very extensive, nor important."

The controversy, however, it is well known, was provoked by Mr. Goldson himself; of which the able and satisfactory answers from every quarter bear ample testimony.

We are told, that Dr. Pearson coincides entirely in opinion with Mr. Goldson, in all his remarks in this chapter. He thinks with Mr. Goldson, that to such writers as have answered him, it is "important to enter into a controversy, but that the issue is of no importance to them." This is not the case with some other writers; who, if they have no character to lose, have certainly one to gain.

---

There is an article immediately subjoined to this Critical Examination, which appears, from its situation and style, to have been manufactured in the same quarter. It is entitled "Instances of supposed Small-pox after Cow-pock." The obvious intention of this article is, not so much to injure the character of vaccination, as that of an individual engaged in the practice. If

If we may judge from the features of this singular performance, it is the offspring of resentment, and disappointed ambition. It is as complete a tissue of falsehood, as ever came from the press. We are told it is confirmed in every essential point by two witnesses. It requires some time to unravel such a fabrication; I pledge myself, however, to prove, by more than two witnesses, that it is a base compound of malice, ignorance, and falsehood.

I am, &c.

*New Street, Hanover Square.* JOHN RING.

